# The role of social media literacy in infodemic management: a systematic review

**DOI:** 10.3389/fdgth.2024.1277499

**Published:** 2024-02-14

**Authors:** Arash Ziapour, Roya Malekzadeh, Fatemeh Darabi, Murat Yıldırım, Nafiseh Montazeri, Neda Kianipour, Nazila Nejhaddadgar

**Affiliations:** ^1^ Cardiovascular Research Center, Health Institute, Imam-Ali Hospital, Kermanshah University of Medical Sciences, Kermanshah, Iran; ^2^ Department of Public Health, Faculty of Health, Health Sciences Research Center, Mazandaran University of Medical Sciences, Sari, Iran; ^3^ Department of Public Health, Asadabad School of Medical Sciences, Asadabad, Iran; ^4^ Department of Psychology, Faculty of Science and Letters, Agri Ibrahim Cecen University, Ağrı, Turkey; ^5^ Department of Social and Educational Sciences, Lebanese American University, Beirut, Lebanon; ^6^ Student Research Committee, Kermanshah University of Medical Sciences, Kermanshah, Iran; ^7^ Department of Public Health, School of Health, Ardabil University of Medical Sciences, Ardabil, Iran

**Keywords:** social media literacy, infodemic, misinformation, health crisis, public health, systematic review

## Abstract

**Background:**

The term infodemic refers to the proliferation of both accurate and inaccurate information that creates a challenge in identifying trustworthy and credible sources. Among the strategies employed to mitigate the impact of the infodemic, social media literacy has emerged as a significant and effective approach. This systematic review examines the role of social media literacy in the management of the infodemic.

**Methods:**

Six databases, including SID, Magiran, Scopus, PubMed, Google Scholar and Web of Science were systematically searched using relevant keywords. We included the relevant publications between 2012 and 2023 in our analysis. To ensure a qualitative assessment of the studies, we used the STROBE and AMSTAR checklists as evaluation tools. The Preferred Reporting Items for Systematic Reviews (PRISMA) guideline was used for the design of this review study. Finally, we organized the studies into groups based on similarities and retrieved and analyzed evidence pertaining to the challenges and opportunities identified.

**Results:**

Eleven papers were included in this study after reviewing the retrieved studies. Five of them examined the effect of social media literacy and health literacy on acceptance of health behaviors. Four studies investigated the role of media literacy in managing misinformation and fake news related to health. Two studies focused on infodemic management and promoting citizen engagement during health crises. Results showed that health-related infodemics are derived from the users' lack of media knowledge, distrust of government service systems, local influencers and peers, rapid circulation of information through mass media messages, weakness of solutions proposed by health care providers, failure to pay attention to the needs of the audience, vertical management, and inconsistency of published messages.

**Conclusion:**

The findings of this study highlight the importance of increasing social media literacy among the general public as a recognized strategy for managing the infodemic. Consequently, it is recommended that relevant organizations and institutions, such as the Ministry of Health, develop targeted training programs to effectively address this need.

## 1 Introduction

The term “infodemic” (1, 2) indicates an extreme volume of information that is typically unreliable and spreads rapidly, especially during the management of a viral epidemic (3, 4). Also, the term infodemic refers to information overload during an epidemic in both digital and physical environments (5, 6). This issue is of particular importance in health-related matters, because the dissemination of misinformation and rumors, particularly through virtual platforms, hinders the effectiveness of public health efforts (6).

The effectiveness of health solutions can be undermined by the creation of confusion and uncertainty among audiences (7, 8) During a pandemic or other health threat, it becomes more important than ever for people to have access to accurate information and they often search for information from multiple sources to increase their awareness (4). However, in the absence of a reliable and transparent source there is a risk that rumors and false information will influence people's understanding of the situation. This risk increases especially through virtual platforms (9).

COVID-19 misinformation and fake news have spread rapidly through social media channels (9). Unfortunately, this increase was accompanied by the dissemination of numerous false and incorrect pieces of information and data. Consequently, it became challenging for audiences to differentiate between reliable information and misleading content, hindering their ability to effectively diagnose and adopt the practical solutions provided by healthcare providers (10). In some cases, this issue prevented people from receiving vaccines or health care and led to a lack of trust in government and public health processes (11).

The Internet is a resource that can be used to access health information. However, it is important to acknowledge that in this context, the spread of fake news and false information tends to outpace the dissemination of fact-based information, contributing to the challenges faced in promoting accurate health information Fake news and false information spread faster and more widely than factual information (12).

Several studies have examined the effects of the infodemic, especially how social behaviors are influenced by such misinformation. In particular, the evaluation of information-related concepts, such as the impact on human lives and societies, the frequency and the most common sources of widespread unreliable data, using comprehensive and evidence-based criteria, has attracted more attention (13–16). In certain countries with a health system centered around family doctors, these doctors serve as the primary gatekeepers of health. In the face of an information epidemic pertaining to health and well-being, they play a crucial role as guides and leaders, actively working to persuade and guide individuals in their societies. In contrast, countries like the United States have a healthcare system that operates on a free market basis, where individuals have the freedom to choose their desired healthcare services (17).

Iran's healthcare system is a combination of these two systems. In Iran, health services (e.g., vaccinations) are the responsibility of the government, and medical services are largely provided in the free market. In such systems, the flow of fake news is much smoother than real news in various fields (18). However, the advent of social media has opened up vast communication platforms where news and information, irrespective of their veracity, have the potential to impact human societies at an unprecedented speed (19).

According to Dutta et al. misinformation has been disseminated through social media (6). The emergence of social media has changed the models of collective action that were based on assumptions about human communication styles. The spread of false information on social networks seems inevitable in times of health and social crises (20).

Social media literacy (SML) is widely regarded as the capacity to critically assess the information that audiences consume or produce, thereby safeguarding them against the dissemination of false information. SML resembles a dietary regimen that astutely attends to the suitability of its constituents, distinguishing between those that are beneficial and those that are detrimental, and determining what ought to be ingested and what ought to be avoided, as well as establishing the foundation for the consumption of each substance. It furnishes individuals with the aptitude to effectively discern and navigate media content, thereby facilitating well-informed decision-making and favorable outcomes (21).

People who have higher SML take a critical stance toward the barrage of media messages. Such individuals act as active elements in society and not only face critical situations against fake news and misinformation but also the expansion of this chain (22). During the health crisis, people with lower SML, tend to use fewer health services and preventive solutions provided by the health system. Consequently, they ended up requiring more emergency services and specialized care during the COVID-19 pandemic (21).

During the COVID-19 pandemic, there was a strong need for reliable sources to address citizens' questions and alleviate their doubts. The initial shock caused by the widespread outbreak of the virus created fertile ground for the emergence of rumors (6, 7, 21).

Improving the SML (which refers to «all media», including television and film, radio and recorded music, print media, the internet and other new digital communication technologies) of media audiences and users of social networks is the most important way to protect people from the spread of misinformation in health crises because SML is a skill that is nurtured by the users' access to reliable and accurate sources that support timely and necessary information (7, 21). Although the field of research on the effectiveness of SML is in its infancy, several studies have tested the role of SML in analyzing media messages and improving one's choices on various health topics (21–23).

Despite the number of studies conducted, the findings have yielded mixed results, leaving several unanswered questions in this field. On the other hand, to date, no comprehensive review of the available research has been conducted in the area of SML interventions with the aim of infodemics management. Furthermore, there has been a lack of systematic analysis to identify the key components that render interventions effective in promoting the accuracy and reliability of information. The understanding of how best to use information in a trustworthy manner remains limited. Therefore, the aim of the present study was to conduct a systematic review that examines the role of SML in effectively addressing the challenges posed by the infodemic. In fact, the researchers want to answer this question with a systematic review, “Is social media literacy effective in managing the infodemic?”

## 2 Materials and methods

This study reviews the evidence on the role of SML in the management of infodemics: The present study is a systematic review performed following the principles of PRISMA. This is a recognized standard for reporting evidence in systematic reviews. It consists of a 27-item checklist to assist reviewers in reporting key characteristics of a systematic review (24). In order to reduce publication bias, all steps of searching, evaluating and selecting articles in addition to data extraction were performed independently by two researchers. Any discrepancies or disagreements were thoroughly discussed until a consensus was reached.

### 2.1 Search strategy

To retrieve English articles, various search strategies were used, including databases such as PubMed, Scopus, Web of Science, and Google Scholar. Additionally, Persian national databases, including the Scientific Information Database (SID) and the Magiran database were searched to include relevant articles in the Persian language. The search did not include any time or language restrictions to ensure that all potentially relevant studies were retrieved. We included the relevant publications up to May 2023. The search for the resources in the mentioned scientific databases was independently conducted by two researchers utilizing the search strategy presented.

#### 2.1.1 Infodemic-related terms [MEDLINE]

Infodemic[keyword] misinformation [keyword] OR disinformation [keyword] OR hoax [keyword] OR deception [Mesh] OR rumor [keyword] OR superstition [keyword, Mesh] OR misconception [keyword] OR misperception [keyword] OR fake news [keyword] OR false news [keyword] OR misleading information [keyword], OR Truthfulness information [keyword] OR SPAM information[keyword] Social advertising [keyword] social messages[keyword]

#### 2.1.2 SML-related terms [MEDLINE]

All studies included SML [keyword] OR electronic skills [Mesh] OR mass media literacy[keyword], information literacy [Mesh] OR internet literacy [Mesh].

Searched using (All “A” terms) AND (All “B” terms)

All studies obtained from the various databases were entered into EndNote X8 software. Initially, duplicate studies were removed. Then, the title and abstract of the studies were reviewed. Following a rigorous assessment based on predefined inclusion and exclusion criteria, unrelated studies were excluded from further analysis. In the eligibility stage, the full text of the studies was reviewed and studies that were not relevant to the purpose of the research were excluded. Finally, articles that met all inclusion criteria were considered for the qualitative assessment.

### 2.2 Study inclusion and evaluation

The search terms were oriented according to the Population, Intervention, Comparison, Results and Study Design (PICOS) approach, the methodology used to select studies for inclusion in the systematic search (24). All English-language studies focusing on the role of SML in the health information epidemic adhered to the following PICOS criteria:

• Population: adult women and men
• Intervention: Not applied
• Comparison: Not applied
• Outcome: Role of SML in the health information epidemic
• Type of study: Cross-sectional studies, Brief overview, Experimental

### 2.3 Inclusion and exclusion criteria

The inclusion criteria were original research articles, availability of the full text of the article, and qualitative studies, while intervention studies, conference abstracts, and letters to the editor were excluded from the study.

### 2.4 Quality assessment

Two different researchers independently assessed the methodological quality of the studies using the cross-sectional studies evaluated with the STROBE checklist (25). The STROBE Statement is a checklist of 22 items relating to the article's title and abstract (item 1), the introduction (items 2 and 3), methods (items 4–12), results (items 13–17), discussion sections (items 18–21), and other information. A higher percentage of items conforming to the guidelines indicates higher methodological quality.

### 2.5 Data extraction

Data extraction was conducted based on a pre-prepared electronic checklist. The checklist consisted of various items, including the first author, year, country, sample size, questionnaire, and study population.

### 2.6 Risk of bias assessment

We followed the recommendations of the Cochrane Handbook to develop risk criteria based on the following: (a) study design (b) participation rate and (c) adjustment for confounding. Therefore, we categorized the risk of bias according to the third criterion only.

### 2.7 Statistical analysis

Two independent researchers extracted the general characteristics of each study and classified them into four distinct categories: (1) reviews evaluating the negative effect of infodemia on health behaviors'; (2) reviews assessing the sources of infodemia; (3) reviews evaluating social media in the spread of fake news, rumors and the emergence of infodemia; and (4) reviews evaluating the relationship of media literacy in controlling invalid news.

We clustered papers based on similar properties related to the stated objective and reported outcomes. We summarized the challenges and opportunities associated with infodemics and misinformation. A third author verified the retrieved data, with another researcher resolving any disagreement between reviewers.

## 3 Results

### 3.1 Study characteristics

**Figure 1:**
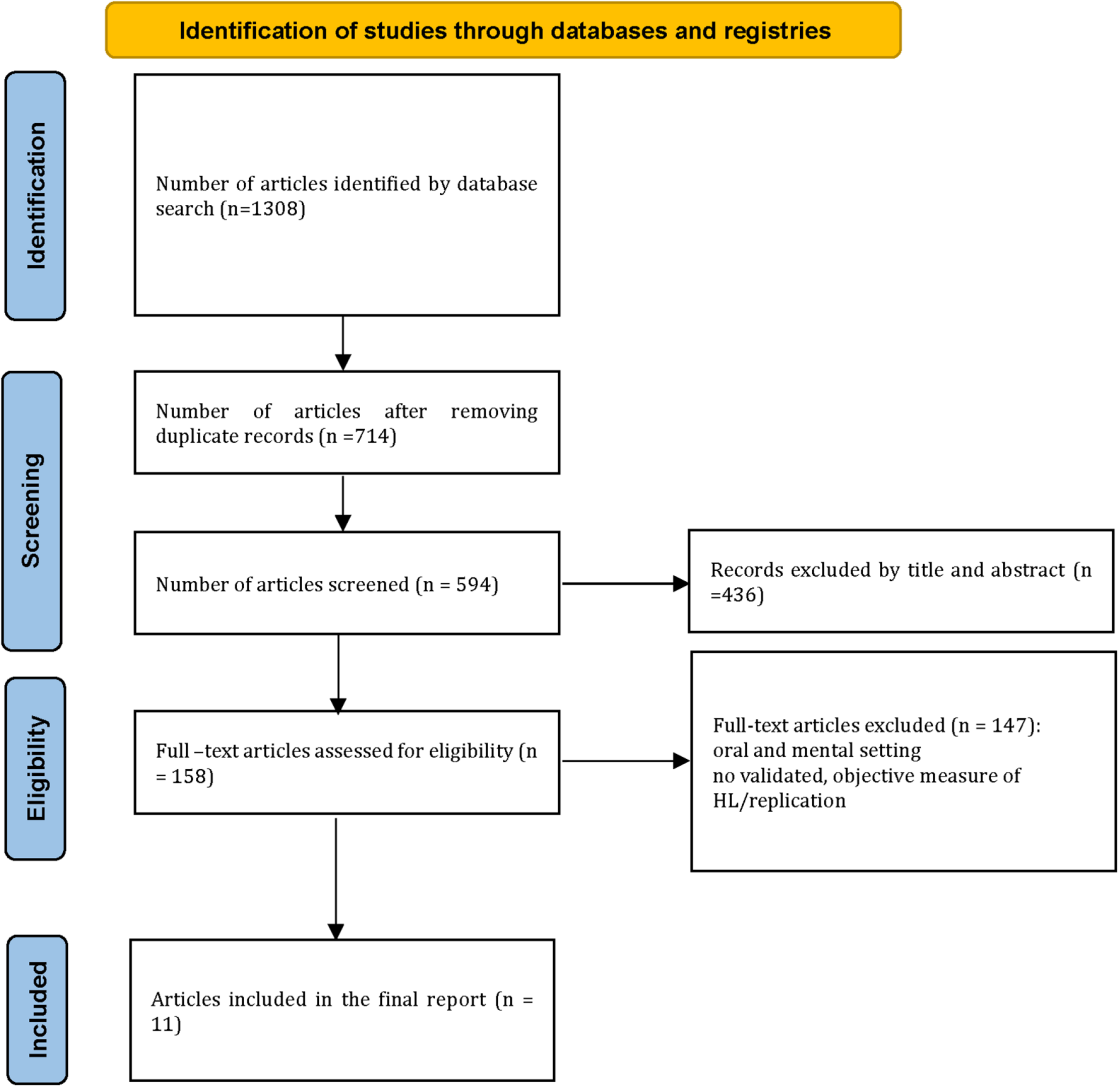
PRISMA chart for the search process.

After the systematic search, a total of 1,308 potentially relevant articles were initially identified from selected databases. After removing 714 duplicate studies, the titles and abstracts of the remaining 594 studies were assessed. Based on this evaluation, 436 studies were excluded, leaving 158 papers for full-text assessment. Out of these, 147 studies were excluded because they did not meet the inclusion criteria. Finally, a total of 11 related articles were included in this study. The study selection process is visually depicted in the PRISMA flowchart, as shown in Figure 1.

### 3.2 Methodological quality

Upon appraisal using the AMSTAR critical domains for systematic reviews, it was observed that 12 out of the 15 reviews (85.7%) obtained critically low-quality scores across several major domains Only two reviews showed a moderate risk of bias in most domains. For cross-sectional studies satisfactory compliance was set at 52%, as has been done elsewhere.

### 3.3 Study characteristics

We included the relevant publications between 2012 and 2023 in our analysis, without restrictions on publication time. The highest publication (3 articles = 27%) was related to China. The largest and smallest sample sizes were related to the study by Liu (2022) with 1,398% and 73% of them being cross-sectional studies. Table 1 shows the overall characteristics of the studies included in the systematic review.

**Table 1:** Summary of the studies included in the systematic review and meta-analysis.

Author and year	Country	Type of study	Research objectives	Sample size	Tools	Main consequence
Inoue et al. (26)	Japan	Cross-sectional study	To identify the relationship between information sources, health literacy, and COVID-19 knowledge in the COVID-19 infodemic	477	Questionnaire	Mass media was the most frequently used source of information, followed by digital media, face-to-face communication, and social media Significant positive correlations were found between media literacy, the number of positive responses to COVID-19 knowledge, and the number of information sources used.
Akbarinejad et al. (27)	Iran	Cross-sectional study	To identify the relationship between media literacy and health literacy among pregnant women	351	Questionnaire	There was a correlation between media literacy and health literacy and all its dimensions except for the critical look at media messages. Providing standard evaluation criteria can help people identify inappropriate information resources so that they can best make their health decisions through media literacy.
Li and Liu (28)	China	Cross-sectional study	To explore the predictive role of social media use on public preventive behaviors	802	Questionnaire- web-based	Social media is an effective tool to promote behaviors to prevent COVID-19 among the public. media literacy is essential for the promotion of individual health and influences the extent to which the public engages in preventive behaviors during a pandemic.
Kim and Jung (29)	South Korea	Cross-sectional study	To examine media use and health information-seeking behavior regarding vaccinations in South Korea	1,367	Web and face-to-face surveys	information about emerging infectious diseases, listening to the radio, and reading the newspaper were associated with increased odds of being vaccinated and Acceptance of health-related behaviors
Liu et al. (30)	China	Cross-sectional study	To examine the underlying mechanisms of the relationship between official social media accounts and the infodemic experienced during the first wave of COVID-19	1,398	Questionnaire	These findings demonstrate the underlying mechanisms of the relationship between official social media accounts and the infodemic. In the context of public health crises, citizens tend to seek information to alleviate uncertainty (e.g., public health, personal and family safety, and recovery efforts) [91]. Our findings show that official social media accounts have controlled the infodemic and increased the social support provided to citizens. In other words, it has alleviated citizens’ uncertainty regarding COVID-19.
Niu et al. (31)	China	Cross-sectional study	To examine health literacy, social media use, and self-efficacy with health information–seeking Intentions among social media users	449	Questionnaire	Health literacy and the use of health-related social media influenced health behavioral goals in social media by influencing self-efficacy and enhancing a sense of participation.
Barati et al. (32)	Iran	Cross-sectional study	To examine the association between COVID-19 media literacy and the fear of COVID-19 and health-related behavior.	300	Questionnaire	COVID-19 media literacy was inadequate among participants. Also, with the publication of contradictory information on social media, distrust among people increased, and distrust prevented people from cooperating with health workers.
Austin et al. (33)	USA	Cross-sectional study	To investigate Media Literacy, Trust of Experts and Flu Vaccine Behaviors Associated with COVID-19 Vaccine Intentions	389	Questionnaire	According to the findings, there was a significant relationship between media literacy and willingness for vaccination. The relationships between the five media literacy skills and the two variables of vaccine type and complications of the vaccine.
Patil et al. (34)	USA	Cross-sectional study	To explore whether COVID-19-related information access, attitudes, and behaviors were associated with health literacy and digital health literacy	1,264	Questionnaire/ Online survey	Media literacy and trust in health experts provided strong counterbalancing influences. Survey-based findings are correlational; thus, predictions are based on theory. Future research should study these relationships with panel data or experimental designs.
Adjin-Tettey (35)	South Africa	Experimental	To assess fake news, disinformation, and misinformation: Experimental evidence for media literacy education	187	Questionnaire	Individuals who received media and information literacy training were able to accurately identify fake stories/information and substitute health behaviors, while untrained individuals could not correctly identify that stories and information were fake. Also, they had poor health behaviors.
Scheibenzube et al. (36)	Germany	Cross-sectional study	To design a training on fake news literacy	102	Questionnaire	Problem-based and participatory online courses on media literacy skills can be suitable learning environments and can serve as a tool to combat fake news illiteracy and infodemic management.

The results showed that health-related infodemics are derived from the users' lack of media knowledge, distrust in government service systems, local influencers and peers, rapid circulation of misinformation through mass media messages, the inadequacy of solutions proposed by health care providers, failure to pay attention to the needs of the audience, vertical management, and contradiction of published messages.

On the other hand, during public health emergencies, mass media can propagate poor-quality information. It is evident that social media has emerged as an ideal channel for the dissemination of rumors, fake news, and general misinformation. Warning, threatening, misleading, shorter messages, the mix of written text and visual images and anecdotal evidence similar to popular culture also have a stronger effect on the dissemination of disinformation and audience acceptance.

In the majority of studies, training and improving skills in using social media and media literacy as a critical method and control strategy is recommended because media literacy plays an important role in reducing the amount of misinformation by limiting false information and directing people to evidence-based websites. For example, the most important cause of vaccine hesitancy messages, fake news, have been published in mass media.

The effect of fake news reduces the participation rate of different age groups in following health behaviors. The next proposed approach to managing the infodemic was community involvement. The findings of the studies highlighted that different age groups interact with the infodemic and the spread of fake news through social media.

## 4 Discussion

This systematic review is the first to evaluate the role of SML in the management of infodemics. Six studies examined the effect of SML on health literacy and acceptance of health behaviors. Among the included studies, four reviewed the role of media literacy in managing misinformation and fake news related to health.

The results of the studies showed that 63.23% of people were able to identify whether the information they received from social media about health-related behaviors was fake news. The findings from our study indicated that a substantial majority of individuals, specifically 71%, particularly in the young and middle-aged age groups, tend to seek information from social media rather than traditional media sources such as TV, radio, or newspapers. Furthermore, the majority of studies that assessed the consequences associated with misinformation on social media pointed to a negative effect on the increased dissemination of poor-quality health-related information during pandemics and diseases.

The publication of unreliable evidence on health issues during a health emergency contributes to the promotion of unscientific and ineffective solutions. The results obtained from the current review showed that SML is an important component of health literacy in promoting preventive strategies related to adherence to health behaviors.

The World Health Organization (23) recommends the implementation of programs aimed at strengthening critical thinking skills. Perazzo et al. (22) reported that higher levels of e-health literacy have led to greater adherence by healthcare workers to those measures designed to prevent and control occupational infections (23). Huhman et al. showed how a mass media campaign increased physical activity, produced positive changes, and prevented negative changes in health-related behaviors (9). Yoğurtcu and Ozturk Haney (10) showed that the development of strategies to improve media literacy among nurses can contribute to the maintenance of health-promoting behaviors among both nurses and their patients (37).

Health information-seeking behavior and media use significantly influence healthy lifestyles, early diagnosis, and sensible disease management (38). Alcott and Gentzkow, indicated that media literacy and e-health literacy are essential for improving people's health and influencing people's preventive behaviors during the pandemic (39). These skills provide the basis for people's participation and cooperation and break the dissemination of this information to other members of the society (38).

When fake news and/or misinformation become part of mainstream news consumption, they can lead to a decrease in trust, and an increase in misinformation (38, 40). Therefore, it is imperative that all information and communication actors are concerned about fake news and misinformation, and contribute to making more people aware of media literacy through proper education (41). For example, during a disease outbreak, the quality of information disseminated by the mass media is critical, as misinformation can also cause panic and lead people to trust harmful or ineffective treatments (42, 43).

According to the results of this study, People with low media literacy pay more attention to news headlines and their emotions in mass media, while technical methods of detecting fake news are more useful for determining the accuracy of information (9).

Infodemic management recognizes the importance of listening to individuals and engaging communities (8). When health authorities and policy-makers understand what issues are capturing people's attention and where there are information gaps, they can respond in real time with high-quality, evidence-based information and recommend interventions.

### 4.1 Limitations and future studies

Regarding the limitations, further cohort investigations might recognize the effects of social media literacy in the management of infodemics among different age and gender groups. The review studies in the past literature have not mentioned the relationship of social media literacy to the role of infodemics in the change and formation of behavior. This current review is noted as a torch because studies related to infodemics are relatively new and require further analysis and review. Researchers can examine the importance of the topic from other angles or may design qualitative studies to minimize the possibility of biased results.

## 5 Conclusion

This study provides evidence that media literacy creates awareness of the consequences of inaccurate information channels and enables information consumers to make informed judgments about information quality. This study highlights the importance of media and information literacy in promoting responsible information sharing. The findings indicate that individuals who possess media literacy skills are more inclined to verify the accuracy of information before sharing it with others. This emphasizes the significance of empowering information consumers with the necessary tools to critically evaluate and assess the reliability of the information they encounter. By partnering with the fields of behavioral science and impact evaluation, in addition to researchers, technical specialists, and social media experts, we can maximize these initiatives' effectiveness and inclusiveness, and get a better sense of their impact during a pandemic.

## Data Availability

The raw data supporting the conclusions of this article will be made available by the authors, without undue reservation.
